# The diagnostic performance of chest computed tomography in the detection of rib fractures in children investigated for suspected physical abuse: a systematic review and meta-analysis

**DOI:** 10.1007/s00330-021-07775-3

**Published:** 2021-03-16

**Authors:** Nasser M Alzahrani, Annmarie Jeanes, Michael Paddock, Farag Shuweihdi, Amaka C. Offiah

**Affiliations:** 1 Diagnostic Radiology Department, College of Applied Medical Sciences, King Abdulaziz University, Jeddah, Saudi Arabia; 2 Academic Unit of Child Health, Department of Oncology and Metabolism, University of Sheffield, Damer Street Building, Western Bank, Sheffield, S10 2TH UK; 3 Department of Paediatric Radiology, Leeds Children’s Hospital, Leeds Teaching Hospitals NHS Trust, Leeds, LS1 3EX UK; 4 Medical Imaging Department, Barnsley Hospital NHS Foundation Trust, Gawber Road, Barnsley, S75 2EP UK; 5 Leeds Institute of Health Sciences, University of Leeds, Leeds, LS2 9NL UK; 6 Radiology Department, Sheffield Children’s NHS Foundation Trust, Western Bank, Sheffield, S10 2TH UK

**Keywords:** Systematic review, Tomography, X-ray computed, Child abuse, Rib fracture, Physical abuse

## Abstract

**Objectives:**

To assess the diagnostic performance of chest CT in the detection of rib fractures in children investigated for suspected physical abuse (SPA).

**Methods:**

Medline, Web of Science and Cochrane databases were searched from January 1980 to April 2020. The QUADAS-2 tool was used to assess the quality of the eligible English-only studies following which a formal narrative synthesis was constructed. Studies reporting true-positive, false-positive, true-negative, and false-negative results were included in the meta-analysis. Overall sensitivity and specificity of chest CT for rib fracture detection were calculated, irrespective of fracture location, and were pooled using a univariate random-effects meta-analysis. The diagnostic accuracy of specific locations along the rib arc (anterior, lateral or posterior) was assessed separately.

**Results:**

Of 242 identified studies, 4 met the inclusion criteria. Of these, 2 were included in the meta-analysis. Chest CT identified 142 rib fractures compared to 79 detected by initial skeletal survey chest radiographs in live children with SPA. Post-mortem CT (PMCT) has low sensitivity (34%) but high specificity (99%) in the detection of rib fractures when compared to the autopsy reference standard. PMCT has low sensitivity (45%, 21% and 42%) but high specificity (99%, 97% and 99%) at anterior, lateral and posterior rib locations, respectively.

**Conclusions:**

Chest CT detects more rib fractures than initial skeletal survey chest radiographs in live children with SPA. PMCT has low sensitivity but high specificity for detecting rib fractures in children investigated for SPA.

**Key Points:**

*• PMCT has low sensitivity (34%) but high specificity (99%) in the detection of rib fractures; extrapolation to CT in live children is difficult.*

*• No studies have compared chest CT with the current accepted practice of initial and follow-up skeletal survey chest radiographs in the detection of rib fractures in live children investigated for SPA.*

**Supplementary Information:**

The online version contains supplementary material available at 10.1007/s00330-021-07775-3.

## Introduction

Physical child abuse is one of the leading causes of child morbidity and mortality worldwide [1, 2]. Physical abuse is defined by the World Health Organization as “acts that cause actual physical harm or have the potential for harm” [3]. In 2013, the prevalence of physical child abuse in the European Region was approximately 23% (44 million children) [4]. Physical abuse is more common in children aged less than 2 years [5, 6], in particular children less than 12 months, who are typically pre-ambulant (those who will typically go on to walk in the future) or non-ambulant (those who will never walk, e.g. wheelchair-bound) who are unable to localise their pain or communicate a history of injury [6, 7].

Following cutaneous injuries, fractures are the second most common finding associated with physical abuse [8]. Rib fractures are strongly associated with physical abuse in infants and young children [9–12] with positive predictive values (PPVs) of 95% and 66% in children under 3 [13] and 4 years of age [14], respectively. Rib fractures are uncommon following accidental trauma in children under the age of 3 years due to the plastic nature of the thoracic cage in this age group [13, 15].

Radiological imaging is an essential tool in the investigation of suspected physical abuse (SPA) in children where the history provided is considered to be inconclusive or incongruent with the clinical examination [16]. International guidelines for skeletal surveys (SkS) have been published by the American College of Radiology (ACR) and the Society for Pediatric Radiology (SPR) [17]; and the Royal College of Radiologists (RCR) and the Society of College of Radiographers (SCoR) endorsed by Royal College of Paediatrics and Child Health (RCPCH) [18], recognised by the European Society of Paediatric Radiology (ESPR) as the gold standard for the investigation of SPA across Europe [19].

These guidelines state that chest radiography is the standard imaging modality employed in the evaluation of the thorax in children suspected of having been physically abused [17, 18]. However, initial chest radiographs (CXR) are often unable to detect acute, non-displaced and incomplete rib fractures [20]. Sanchez et al [21] found that 17% of rib fractures studied (total 105 fractures) in cases of SPA were not visualised on CXR. Recent studies have shown that oblique projections of the thorax have improved the detection of rib fractures in SPA [22–24], both on initial (acute) and follow-up (healing) SkS. However, even with additional oblique views of the thorax, acute rib fractures are often difficult to detect [6, 25] due to fracture lines potentially being masked by overlying lung and vascular markings [26, 27], in addition to being superimposed over other anatomical structures [20]. The rationale behind the inclusion of the CXR as part of the follow-up SkS 11–14 days after the initial SkS relates to the formation of callus associated with rib fracture healing [7], thereby increasing their conspicuity and improving the detection of those fractures not visualised on the initial SkS [28–30].

It has been shown that chest computed tomography (CT) improves the detection of acute and healing rib fractures in live [31] and post-mortem (PM) children [26]. Unlike CXR, chest CT can more accurately diagnose acute rib fractures and could offer immediate evidence of inflicted injury. The use of chest CT may avoid follow-up CXR at possibly comparable radiation doses [21, 32].

We systematically reviewed the available evidence concerning the diagnostic performance of chest CT in the detection of rib fractures in live and post-mortem children with suspected or confirmed physical abuse in comparison to the established reference standard of CXR and/or autopsy. The primary objective was to evaluate whether the diagnostic performance of chest CT is comparable to other established standard methods of diagnosing rib fractures in children with SPA. The second objective was to assess the diagnostic accuracy (sensitivity and specificity) of chest CT in the detection of rib fractures at different anatomical locations (anterior, lateral and posterior) along the rib arc.

## Methods

The study protocol for this systematic review was registered on PROSPERO (registration number: CRD42020179550) and the study was conducted in accordance with the Preferred Reporting Items for Systematic Reviews and Meta-Analyses (PRISMA) statement guidelines [33].

### Data sources and searches

Medline, Web of Science and Cochrane databases were searched for eligible articles published in English between January 1980 and April 2020. The search strategy and terms used are provided in the Electronic Supplementary Material. The reference lists of all included articles were searched for additional articles not captured in the initial search.

### Study selection

Study inclusion and exclusion criteria were developed based on population, intervention, comparator and outcomes (PICO) criteria:

- P: Children (0–18 years [34]) with suspected or known rib fractures resulting from confirmed or SPA
- I: Chest CT imaging
- C: CXR and/or autopsy
- O: Diagnostic accuracy of CT in the detection of rib fractures in children resulting from confirmed or SPA

Studies satisfying the following criteria were included: (1) study participants aged up to 18 years old with suspected or known rib fractures resulting from physical abuse; (2) chest CT used as a diagnostic tool to detect rib fractures; (3) the diagnostic performance of chest CT compared to the reference standards of either CXR and/or autopsy was reported; (4) for meta-analysis, the absolute numbers of true positives (TP), true negatives (TN), false positives (FP), and false negatives (FN) were reported or could be derived to calculate sensitivity and specificity. We excluded (1) studies performed on animals and/or phantoms; (2) studies where the manuscript body was not in English; (3) case reports, review articles, editorial/comment papers and abstracts of conference meetings. Following the removal of duplicates, study titles and abstracts were screened by one reviewer (N.M.A.). Full-text screening of potentially eligible studies was then performed to further confirm eligibility. A consensus opinion was sought by two reviewers (A.J. and A.C.O.) to resolve any uncertainties.

### Data extraction and quality assessment

Data were extracted from the eligible studies using a predesigned data collection form which included author; publication year; study design; sample size and number of ribs reviewed; mean age; reference test; CT imaging protocol; the time interval between the reference standard and CT imaging and the study primary outcome. For this systematic review, the acceptable time intervals between the index test (chest CT) and the reference standards were ≤ 48 h (CXR) and/or ≤ 1 week (autopsy). For meta-analyses, TP, TN, FP and FN were derived and pooled from the included studies to assess the diagnostic performance of chest CT. Four reviewers (N.M.A., A.J., M.P., A.C.O.) independently assessed the quality of the included studies using the Quality Assessment of Diagnostic Accuracy Studies 2 (QUADAS-2) tool [35]. Discrepancies between reviewers were resolved by consensus.

### Data synthesis and analysis

We performed a formal narrative synthesis of the findings from the eligible studies which included a summary of the study characteristics and outcome measures regarding the diagnostic performance of chest CT. To perform a meta-analysis, summary measures of TP, FP, TN and FN rates were calculated for individual studies to express the diagnostic performance of chest CT imaging in terms of sensitivity, specificity, positive predictive value (PPV) and negative predictive value (NPV) with respect to the detection of rib fractures. To assess the overall diagnostic performance of chest CT against the reference standards, the diagnostic accuracy measures reported for all rib fractures in each study were pooled with those that utilised the same reference standard. Separately, to assess the diagnostic accuracy of chest CT in the detection of rib fractures based on specific locations along the rib arc (anterior, lateral and posterior), the diagnostic accuracy measures reported in studies were pooled with those using the same reference standard for each location. A meta-analysis was performed when at least two studies met the criteria.

Forest plots estimating the sensitivity and specificity with 95% confidence intervals (CIs) were created using a univariate random-effects model. Moreover, a receiver operator characteristic (ROC) curve was generated to calculate the area under the curve (AUC).

Heterogeneity between studies included in the meta-analysis was assessed using the *I*^2^ statistic with heterogeneity categorised by low (0–40%), moderate (50–75%), and high (> 75%) [36]. Meta-analyses were performed using STATA 14 package metandi (STATA Corporation).

## Results

### Search strategy

**Fig. 1:**
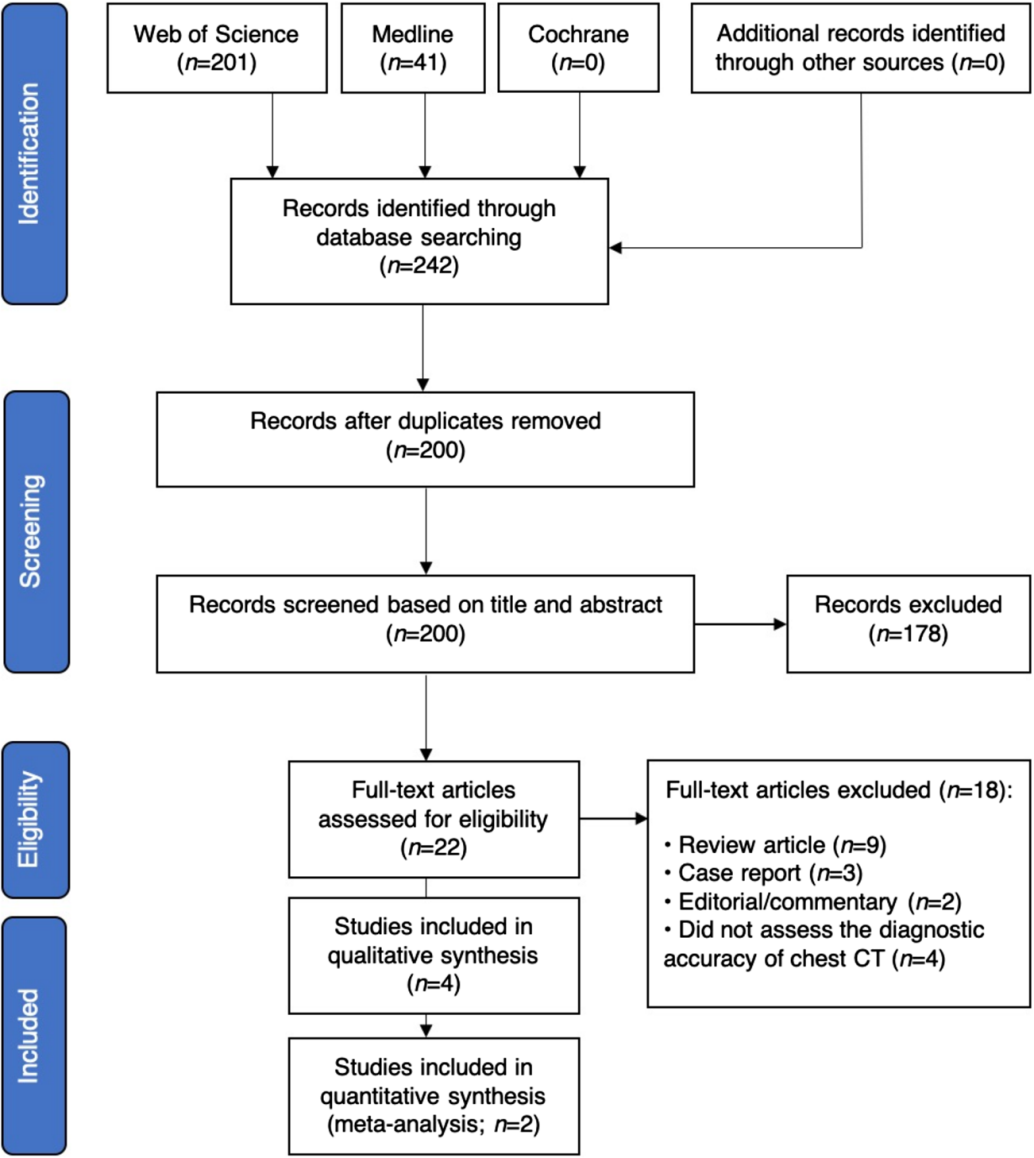
Flowchart of the study selection process

Of 242 articles identified, 42 duplicates and 178 articles were excluded on the basis of title and abstract. Of the 22 full-text articles extracted, 18 did not fulfil the inclusion criteria. The remaining 4 articles were included for qualitative synthesis. Of these, two studies [21, 31] were excluded from the meta-analysis because the TP, TN, FP and FN values were not reported or could not be derived. Figure 1 illustrates the search strategy and study selection process.

### Study characteristics and quality assessment

The characteristics and main findings of the included studies are summarised in Table 1. These observational retrospective studies included a total of 109 children. The mean age per study ranged from 2.5 to 12 months, with a mean age of 6 months across all studies. The reference standards against which chest CT was compared were CXR from initial SkS in two studies (time interval 1 day) and autopsy in the remaining two (time interval 0 to 5 days, median 2 days). The tube voltage (kilovolts, kV) and current (milliampere, mA) settings for chest post-mortem CT (PMCT) were 120 kV and 200–355 mA and 100 kV and 15 mA in live children, respectively.

**Table 1:** Characteristics and main findings of the included studies

Study	1	2	3	4
Author (year)	Wootton-Gorges et al (2008) [31]	Hong et al (2011) [26]	Sanchez et al (2018) [21]	Shelmerdine et al (2018) [25]
Study design (recruitment start and end dates)	Retrospective (between 1999 and 2004)	Retrospective (between January 2007 and October 2009)	Retrospective (between January 2008 and January 2012)	Retrospective (between January 2012 and January 2017)
Sample size/number of reviewed ribs	12/225 ribs	56/1318 ribs reviewed at primary (clinical) interpretation and 298 ribs reviewed at research radiologist’s interpretation	16 (only 5 of whom had both CXR and chest CT) / Not reported	25/600 ribs (12 pairs of ribs in 25 children)
Mean age (range)	Mean age 2.5 months (1.2 to 5.6 months)	Mean age 12 months (8 days to ~93 months); 46 children < 24 months)	Mean age 6 months (1 to 11 months)	Median age 4 months (17 days to 7 years)
Reference test	Initial CXR (AP and lateral projections)	Autopsy	Initial CXR (AP, lateral, right and left oblique projections)	Autopsy
CT imaging protocol	Not reported	• Either 8 multi-slice helical CT (GE), 128-slice MDCT (GE) or 16-slice CT (Philips) • 120 kV, 320 and 355 mA, 0.53 and 0.93 mm pitch, rotation time 0.75 and 0.80 s • Slice thickness reconstruction 0.80–0.62 mm • 3D reconstruction	• 100 kV, 15 mA, 1 mm pitch, rotation time 0.5 s • Adaptive iterative reconstruction blend of 20%	• 64-slice MDCT (Siemens) • Collimation 0.62 mm • 120 kV, 200–350 mA,1 mm pitch • Slice thickness reconstruction (1 mm)
Time interval between the reference standard and CT imaging	1 day	Not reported	1 day	Median 2 days (range 0 to 5 days)
The study primary outcome	• Chest CT = 131 rib fractures • CXR = 79 rib fractures • CT more sensitive in the detection of rib fractures based upon anatomical position than CXR in the detection of rib fractures at all anatomical positions (*p* < 0.01), except in lateral location • Two radiologists reviewed the chest CT studies	• 101 rib fractures at autopsy (standard) •Two image interpretation methods were used for the detection rib fractures by fracture locations: A. Primary (clinical) interpretation=chest CT showed sensitivity 51.5% and specificity 99.7% for detecting rib fractures by fracture locations • PM CXR for detection of rib fractures: at specified locations=sensitivity 28.9% and specificity 99.9%; the sensitivity and specificity of PM CXR at specific locations along rib arc* was 8% and 99% at anterior; 80% and 100% at lateral; 29% and 98% at posterior B. Radiologist interpretation = 13 children: 12 with rib fractures at autopsy and 1 false positive at chest radiograph primary interpretation; chest CT showed sensitivity 85.1% and specificity 99.4% for detecting rib fractures by fracture locations • Two radiologists reviewed chest CT (3 studies reviewed by one radiologist) at primary (clinical) interpretation; radiologist interpretation performed by one radiologist only	• Chest CT = 11 rib fractures all missed on initial CXR •7 rib fractures identified on follow-up† CXR (1–2 weeks) in patients who did not have chest CT •The number of radiologists who reviewed chest CT was not reported	• 136 rib fractures at autopsy (standard) •Chest CT for detection of rib fractures: at specified locations=sensitivity 44.9% and specificity 97% •PM CXR for detection of rib fractures: at specified locations=sensitivity 13.5% and specificity 97.9%; the sensitivity and specificity of PM CXR at specific locations along rib arc was 15% and 97% at anterior, 0.8% and 98% at lateral, 27% and 98% at posterior, respectively •Thirty-five radiologists reviewed the CT chest studies and thirty-eight reviewed the CXR studies

*The location of the rib fractures was divided into three segments: anterior fracture includes costochondral, anterior and anterolateral; posterior fracture includes costovertebral, posterior and posterolateral; and lateral. Calculated as follows [anterior = (anterior + anterolateral) / 2]

^†^Follow-up skeletal survey

*3D*, three-dimensional; *AP*, anteroposterior; *CT*, computed tomography; *CXR*, chest radiographs; *GE*, General Electric; *kV*, kilovolts; *mA*, milliampere; *MDCT*, multidetector computed tomography; *mm*, millimeter; *PM*, post-mortem; *s*, seconds

Table 2 summarises the results of the quality assessment of the 4 included studies. In the patient selection domain, three studies [21, 25, 31] were assessed as having an unclear risk of bias due to poor sampling procedure reporting. One study [21] was scored as having an unclear risk of bias in the index test domain as it was unclear whether the chest CT results were interpreted without knowledge of reference standard results. In the reference standard domain, two studies [21, 26] were judged as having an unclear risk of bias because they did not offer information on whether the reference standard was interpreted without knowledge of the results of the index test. Regarding applicability concerns, one study [21] had a high risk of concern in the index test domain because chest CT was only performed in children with normal initial CXR: this affects the diagnostic accuracy as all children with positive and negative initial CXR were supposed to have chest CT. Additionally, one study [31] had an unclear risk of concern in the patient selection domain because information about the clinical indication for patients who underwent CT examinations was not provided.

**Table 2:** Quality assessment of included studies

Study	Risk of bias				Applicability concerns		
	Patient selection	Index test	Reference standard	Flow and timing	Patient selection	Index test	Reference standard
Hong et al [26]	1	1	2	1	1	1	1
Sanchez et al [21]	2	2	2	1	1	3	1
Shelmerdine et al [25]	2	1	1	1	1	1	1
Wootton-Gorges et al [31]	2	1	1	1	2	1	1

Low risk = 1; unclear risk = 2; high risk = 3

### Diagnostic performance of chest CT

Two retrospective studies [21, 31] demonstrated superior diagnostic accuracy of chest CT in the detection of rib fractures compared to initial CXR. Sanchez et al [21] reported that chest CT detected 11 rib fractures that were missed on initial CXR. Wootton-Gorges et al [31] observed 131 rib fractures on chest CT compared to 79 on CXR. Additionally, Wootton-Gorges et al [31] reported that chest CT performed better in detecting rib fractures along the rib arc (*p* < 0.01), except for lateral locations.

The retrospective studies by Hong et al [26] and Shelmerdine et al [25] compared the diagnostic accuracy of chest CT to autopsy in the detection of rib fractures. Chest CT reported sensitivity (51.5% and 44.9%, respectively) and specificity (99.7% and 97.0%, respectively) in the detection of rib fractures at specific locations. Hong et al [26] noted that the sensitivity for detecting rib fractures at specific locations increased from 51.5% at primary (clinical) interpretation to 85.1% following radiologist interpretation.

**Fig. 2:**
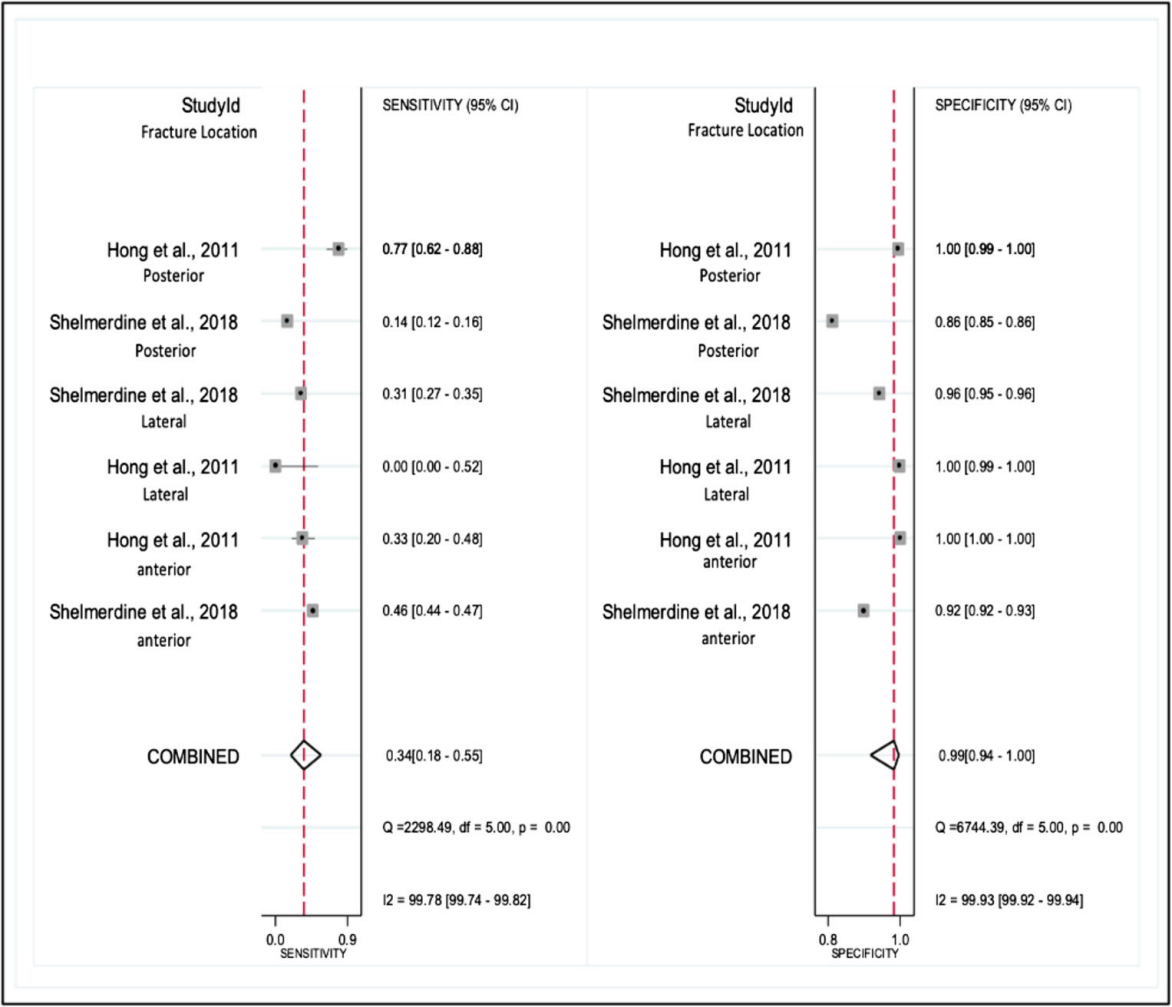
Forest plot of the overall sensitivity and specificity with 95% confidence intervals of chest CT in the detection of rib fractures

We grouped two studies (*n* = 81 children) regarding the diagnostic performance of chest CT as compared to autopsy in the detection of rib fractures: Table 3 presents the sensitivity and specificity of chest CT. Forest plots for overall chest CT diagnostic performance demonstrated sensitivity 34% (95% CI 18–55%) and specificity 99% (95% CI 94–100%) (Fig. 2) and PPV 95% (95% CI 91–100%), NPV 59% (95% CI 58–61%) and AUC 79% (95% CI 75–82%). Significant heterogeneity existed for both sensitivity and specificity (*I*^*2*^ = 99%). The pooled diagnostic sensitivity and specificity of chest CT in the detection of rib fractures along the rib arc were 45% and 99% at anterior locations, 21% and 99% at lateral locations and 42% and 97% at posterior locations, respectively.

**Table 3:** Diagnostic performance measures (sensitivity and specificity) of chest CT in the detection rib fractures

Study	Reference test	Fracture location along rib arc*	Number of ribs included in analysis	Number of observations	Number of rib fractures identified by the reference test	TP	TN	FP	FN	Sensitivity %	Specificity %
Hong et al [26]		Anterior			49	16	2587	3	33	33	100
	Autopsy	Lateral	1318	N/A	5	0	1313	3	5	0	100
		Posterior			47	36	2589	12	11	77	100
Shelmerdine et al [25]		Anterior			111	1769	15 783	1332	2116	46	92
	Autopsy	Lateral	600	21 000^†^	10	164	19 574	901	361	31	96
		Posterior			15	164	6479	1081	1011	14	86

*The location of the rib fractures was divided into three segments: anterior fracture includes = costochondral, anterior and anterolateral; posterior fracture includes = costovertebral, posterior and posterolateral; and lateral

^†^Calculated as 25 cases × 24 ribs × 35 reporters

*FN*, false negative; *FP*, false positive; *N/A*, not applicable; *TN*, true negative; *TP*, true positive

## Discussion

This systematic review and meta-analysis provides an overview of the diagnostic performance of chest CT in the detection of rib fractures in children with SPA. This study demonstrates that chest CT detects more rib fractures than initial CXR. The overall diagnostic performance of PMCT is 34% sensitivity and 99% specificity in the detection of rib fractures when compared to autopsy, whilst PM CXR showed a sensitivity of 13.5% and 28% and specificity of 97% and 99% [25, 26]. Overall, PMCT exhibited the lowest sensitivity for lateral rib fracture locations, with sensitivities of 45%, 21% and 42% at anterior, lateral and posterior locations, respectively. The diagnostic performance of PM CXR at specific rib fracture locations reported in two of the included studies [25, 26] was sensitivity: 8% and 15.8% at anterior, 80% and 0.8% at lateral and 29% and 27% at posterior rib fracture locations, respectively. PM CXR pooled sensitivities, specificities and meta-analyses were not performed due to the different projections obtained in the included studies: 3 projections (AP, right and left obliques) were utilised by Shelmerdine et al [25] whereas only 2 projections (AP and lateral) were used by Hong et al [26]. It has been reported by Hong et al [26] that PM CXR missed 42 anterior rib fractures whilst 27 anterior rib fractures were missed on CT in children who underwent cardiopulmonary resuscitation.

Notably, chest CT detected more rib fractures than initial CXR in abused live children. In 2 studies, chest CT identified 142 rib fractures compared to 79 detected by initial CXR [21, 31]. This is because CT provides high-resolution cross-sectional images of the thoracic cage with volumetric and multi-planar reconstructions [37] eliminating the contributing factors which obscure rib fractures on CXR; in particular, acute and/or non-displaced fracture where callus formation, indicative of healing, is not present [7].

The overall low sensitivity (34%) and high specificity (99%) of PMCT in this systematic review are consistent with the results of a study validating PMCT against autopsy in the detection of rib fractures in adults (low sensitivity of 58% and high specificity of 97%) [38]. A possible explanation of this low sensitivity is that PMCT may not accurately detect reattached rib fracture edges on autopsy [38]. Moreover, autopsy is not a perfect reference standard due to its fallibility with respect to partial rib fractures which are more easily detected by PMCT [38]. Interestingly, Hong et al [26] observed that PMCT sensitivity increases from 51.1% at primary (clinical) interpretation to 85.1% at radiologist interpretation: not an unexpected result given that the interpretation was performed by an experienced radiologist (22 years’ experience). High heterogeneity in reported sensitivity and specificity was observed among the included studies which could be secondary to the differences in radiologists’ experience and the imaging protocols employed.

Concerns regarding the relatively high radiation exposure have traditionally made chest CT a less desirable option in the routine clinical investigation of SPA in live children [39]. It is well-documented that children are more vulnerable to the effects of ionising radiation and potential future risk of radiation-induced cancer than adults, in addition to a greater time over which the consequences of radiation exposure may be borne out [40–43]. However, adjusting the scanning parameters (e.g. mA, kV and pitch) and the use of iterative reconstruction reduces the radiation dose and the consequent potential risk and concerns regarding radiation-induced cancers [44, 45]. Recently, a developed low-dose chest CT (LDCT) protocol for the detection of rib fractures in children with SPA employed radiation doses approaching those of standard CXR without compromising diagnostic quality [21, 32].

The risk of exposure to ionising radiation in the case of chest CT should be balanced against the risk of missed diagnoses of physical child abuse, in particular, missing occult acute rib fractures on initial SkS CXR [21]. Given that follow-up SkS imaging is not guaranteed (as children are reliant on their caregiver/parent to return them to the hospital for follow-up imaging), children may remain in an abusive environment, which risks sustaining a further, potentially fatal, injury [46]. Chest CT demonstrates a higher sensitivity in the detection of acute rib fractures in live children who may have been abused, thus potentially rendering the follow-up SkS CXR redundant. Moreover, this would result in an overall reduction in radiation burden if LDCT protocols are utilised and may facilitate more prompt and appropriate management in cases of child protection.

This study has several limitations. First, a small number of studies (*n *= 2) were included in the meta-analysis which is insufficient to accurately evaluate the diagnostic performance of PMCT in the detection of rib fractures. Therefore, the results of the meta-analysis should be interpreted cautiously. Notably, the results of this meta-analysis should be restricted to the diagnostic performance of chest CT in PM children. The image quality of chest PMCT examinations may be higher than in live children (given that dose restrictions are not a consideration) which may increase its diagnostic accuracy. Second, all included studies were retrospective. Third, whilst the reference standards used to assess the accuracy of the chest CT in the detection of rib fractures and skill of the radiologist reading them are those used in current clinical practice, they themselves are imperfect. Fourth, although physical abuse is not common over the age of 2 years, our search criteria included participant age up to 18 years to ensure that we captured all relevant papers. In total, 13 children (11.9%) were over the age of 2 years, with a mean age of 6 months across all 4 papers; therefore, we believe the results of this review are applicable to cases of SPA. Finally, this systematic review might be prone to publication bias given that the literature search was restricted to English language studies only.

Although chest CT shows promising results in the detection of acute and healing rib fractures, further research is required to better elucidate its diagnostic performance. Ideally, the diagnostic accuracy of chest CT (compared to a reference standard of initial and follow-up CXR from SkS) should be evaluated in a prospective study with a large cohort of live children. Additionally, to adhere to the ALARA principle, evaluation of a LDCT chest protocol to reduce exposure to ionising radiation could be conducted prior to formal implementation in clinical practice.

In conclusion, the diagnostic performance of chest CT for detecting rib fractures in children suspected of having been physically abused has not previously been systematically evaluated in the literature. Chest CT detects more rib fractures than initial CXR in children with SPA. PMCT has a low sensitivity but high specificity for detecting rib fractures (especially in lateral locations) compared to autopsy.

## Supplementary information

ESM 1 (DOCX 26 kb)

## Abbreviations

3D: Three-dimensional
ACR: American College of Radiology
ALARA: As low as reasonably achievable
CI: Confidence interval
CXR: Chest radiographs
ESPR: European Society of Paediatric Radiology
FN: False negative
FP: False positive
LDCT: Low-dose computed tomography
MDCT: Multidetector computed tomography
mSv: millisievert
NPV: Negative predictive value
PMCT: Post-mortem CT
PPV: Positive predictive value
PRISMA: Preferred Reporting Items for Systematic Review and Meta-Analysis
QUADAS-2: Quality Assessment of Diagnostic Accuracy Studies 2
RCPCH: Royal College of Paediatrics and Child Health
RCR: Royal College of Radiologists
SCoR: Society and College of Radiographers
SkS: Skeletal survey
SPA: Suspected physical abuse
SPR: Society for Pediatric Radiology
TN: True negative
TP: True positive

## Declarations

### Guarantor

The scientific guarantor of this publication is Professor Amaka C Offiah.

### Statistics and biometry

Farag Shuweihdi is a research fellow and a Medical Statistician at the Leeds Institute of Health Sciences, University of Leeds, Leeds.

### Methodology

• Systematic review

• Meta-analysis
