# Supplementary material for: The diagnostic performance of chest computed tomography in the detection of rib fractures in children investigated for suspected physical abuse: a systematic review and meta-analysis
Source: Eur Radiol. 2021 Mar 16;31(9):7088–97. doi: 10.1007/s00330-021-07775-3 (PMC8379101; doi:10.1007/s00330-021-07775-3)
Supplement: Supplementary file 1 — (DOCX 26 kb) [file 330_2021_7775_MOESM1_ESM.docx]

**ELECTRONIC SUPPLEMENTARY MATERIAL (ESM)**

**Appendix**

1. Search strategy

A.1. Medline

| **#** | **Searches** | **Results** |
| --- | --- | --- |
| 1 | Infant/ | 783193 |
| 2 | (paediatric* or pediatric*).mp. | 395627 |
| 3 | (child* or babies or baby or toddler*).mp. | 2457496 |
| 4 | (Adolescent or Young*).mp. | 2875468 |
| 5 | 1 or 2 or 3 or 4 | 4437538 |
| 6 | Child Abuse/ | 21907 |
| 7 | (battered child or shaken baby or battered baby).mp. | 1707 |
| 8 | (non-accidental trauma or nonaccidental trauma).mp. | 380 |
| 9 | (non-accidental injur* or nonaccidental injur* or NAT).mp. | 7578 |
| 10 | Physical Abuse/ | 585 |
| 11 | (Occult skeletal trauma or Occult injur*).mp. | 257 |
| 12 | (Inflicted injur* or unexplained inju*).mp. | 872 |
| 13 | 6 or 7 or 8 or 9 or 10 or 11 or 12 | 31265 |
| 14 | rib fractur*.mp. | 4547 |
| 15 | Costal fractur*.mp. | 67 |
| 16 | (thoracic Injur* or Chest Injur*).mp. | 14176 |
| 17 | Rib Fractures/ | 3040 |
| 18 | 14 or 15 or 16 or 17 | 17558 |
| 19 | Tomography, X-Ray Computed/ | 375378 |
| 20 | Thoracic CT.mp. | 1400 |
| 21 | Chest CT.mp. | 7177 |
| 22 | CT.mp. | 336347 |
| 23 | 19 or 20 or 21 or 22 | 563591 |
| 24 | 5 and 13 and 18 and 23 | 47 |
| 25 | limit 24 to (english language and yr="1980 -Current") | 41 |

A.2. Web of Science database

| **#** | **Searches** | **Results** |
| --- | --- | --- |
| 1 | TS=(Infant* OR Newborn) | 1,532,622 |
| 2 | TS=(paediatric* OR pediatric*) | 1,814,713 |
| 3 | TS=(child* or (babies or baby) or toddler*) | 3,570,524 |
| 4 | TS=(Adolescent or Young*) | 3,682,274 |
| 5 | #4 OR #3 OR #2 OR #1 | 6,753,712 |
| 6 | TS=(Child* Abuse) | 95,299 |
| 7 | TS=(battered child* OR shaken baby OR battered baby) | 5,606 |
| 8 | (TS=(non-accidental trauma or nonaccidental trauma) | 1,126 |
| 9 | Ts=(non-accidental injur* or nonaccidental injur* or NAT) | 45,021 |
| 10 | Ts=Physical Abuse | 39,537 |
| 11 | TS=(Occult skeletal trauma or Occult injur*) | 5,604 |
| 12 | TS=(unexplained injur*) | 3,627 |
| 13 | TS=(Inflicted injur*) | 7,201 |
| 14 | #13 OR #12 OR #11 OR #10 OR #9 OR #8 OR #7 OR #6 | 176,374 |
| 15 | Ts=rib fractur* | 9,867 |
| 16 | ts=thoracic Injur* | 46,280 |
| 17 | ts=Costal fractur* | 388 |
| 18 | TS=(thoracic Injur* or Chest Injur*) | 68,448 |
| 19 | #18 OR #17 OR #16 OR #15 | 75,721 |
| 20 | TS=(Computed tomography OR CT) | 1,159,724 |
| 21 | ts=chest CT | 41,455 |
| 22 | ts=Thoracic CT | 26,476 |
| 23 | #22 OR #21 OR #20 | 1,159,724 |
| 24 | #23 AND #19 AND #14 AND #5 | 220 |
| 25 | #23 AND #19 AND #14 AND #5  Refined by: LANGUAGES: ( ENGLISH ) Timespan=1980-2020 | 201 |

A.3. Cochrane databases

| **#** | **Searches** | **Results** |
| --- | --- | --- |
| 1 | MeSH descriptor: [Infant] explode all trees | 16136 |
| 2 | (("paediatric*" or "pediatric*")):ti,ab,kw | 31299 |
| 3 | child* or babies or baby or toddler*):ti,ab,kw | 152842 |
| 4 | (Adolescent or Young*):ti,ab,kw | 201410 |
| 5 | #1 OR #2 OR #3 OR #4 | 312145 |
| 6 | MeSH descriptor: [Child Abuse] explode all trees | 503 |
| 7 | ("battered child*" or "shaken baby" or "battered baby"):ti,ab,kw | 19 |
| 8 | ("non-accidental trauma" or "nonaccidental trauma"):ti,ab,kw | 5 |
| 9 | ("non-accidental injur*" or "nonaccidental injur*" or | 253 |
| 10 | MeSH descriptor: [Physical Abuse] explode all trees | 16 |
| 11 | ("Occult skeletal trauma" or "Occult injur*"):ti,ab,kw | 0 |
| 12 | ("Inflicted injur*" or "unexplained inju*"):ti,ab,kw | 0 |
| 13 | #6 OR #7 OR #8 OR #9 OR #10 OR #11 OR #12 | 787 |
| 14 | ("rib fractur*"):ti,ab,kw | 282 |
| 15 | ("Costal fractur*"):ti,ab,kw | 4 |
| 16 | (("thoracic Injur*" or "Chest Injur*")):ti,ab,kw | 1 |
| 17 | MeSH descriptor: [Rib Fractures] explode all trees | 79 |
| 18 | #14 OR #15 OR #16 OR #17 | 283 |
| 19 | MeSH descriptor: [Tomography, X-Ray Computed] explode all trees | 4826 |
| 20 | ("Thoracic CT"):ti,ab,kw | 75 |
| 21 | ("Chest CT"):ti,ab,kw | 466 |
| 22 | (CT):ti,ab,kw | 74831 |
| 23 | #19 OR #20 OR #21 OR #22 | 76872 |
| 24 | #23 AND #18 AND #13 AND #5 | 0 |
